# Frey’s syndrome in an infant misdiagnosed as food allergy

**DOI:** 10.1186/s13223-026-01015-3

**Published:** 2026-02-15

**Authors:** Melanie Semaan, Sheikha Alkhuder, Samira Jeimy

**Affiliations:** Division of Clinical Immunology and Allergy, Department of Medicine, Western University, 268 Grosvenor Street, London, ON N6A 4V2 Canada

**Keywords:** Food allergy, Frey syndrome, Auriculotemporal syndrome

## Abstract

**Background:**

Frey’s syndrome (auriculotemporal syndrome) is characterized by gustatory flushing and sweating in the distribution of the auriculotemporal nerve. Although classically described after parotid surgery in adults, congenital and idiopathic forms are increasingly recognized in infants. Because symptoms occur during feeding, the condition can closely mimic IgE-mediated food allergy, leading to unnecessary dietary restrictions and anxiety.

**Case presentation:**

We report an 8-month-old boy referred for evaluation of suspected multiple food allergies after several emergency department visits for feeding-associated facial redness, interpreted by caregivers as “hives”, and accompanied by significant parental anxiety and progressive dietary avoidance. On detailed reassessment, reactions were found to consist of localized, unilateral flushing and sweating of the right cheek, without urticaria or systemic features. Skin prick testing and serum-specific IgE testing to common food allergens were negative. During a supervised baked milk oral food challenge, a similar unilateral cutaneous reaction was observed, confirming the diagnosis of Frey’s syndrome. A home-recorded video demonstrated comparable findings during feeding with formula.

**Discussion:**

Frey’s syndrome is an important and under-recognized mimic of food allergy in infancy. Careful attention to laterality, absence of systemic involvement, and reproducibility across foods can prevent misdiagnosis, reduce unnecessary dietary restriction, and alleviate family anxiety.

**Conclusion:**

Frey’s syndrome should be considered in infants with reproducible unilateral flushing during feeding. Awareness among allergists can prevent misdiagnosis, reduce unnecessary food avoidance, and support safe nutritional and developmental outcomes.

## Introduction

Food allergy evaluation in infancy is a frequent referral to allergy clinics. Clinical histories often involve nonspecific cutaneous findings such as flushing or erythema. When temporally associated with feeding, these are frequently interpreted as IgE-mediated food allergy, leading to unnecessary testing and inappropriate dietary avoidance.

Frey’s syndrome, or auriculotemporal syndrome, manifests as unilateral flushing and sweating triggered by gustatory stimuli. While well documented in adults after parotidectomy, pediatric presentations, including congenital and idiopathic forms, are less well known. Because the syndrome manifests during eating, it can be mistaken for food allergy in children, particularly infants.

This case adds to the growing literature on infantile Frey’s syndrome and highlights the importance of recognizing its distinguishing features in allergy practice.

## Case presentation

An 8-month-old boy was referred to the allergy clinic for evaluation of suspected multiple food allergies. At referral, the family described immediate facial “hives” following milk, soy, and solid food (peanut, wheat, egg) ingestion, prompting several emergency department visits, progressive dietary avoidance, and significant parental anxiety surrounding feeding. The history provided emphasized acute onset reactions temporally associated with feeding, raising concern for IgE-mediated food allergy.

On detailed reassessment, the episodes were found to consist of flushing and diaphoresis, without angioedema, respiratory symptoms, gastrointestinal involvement, or other systemic features. Reactions were reproducible with feeding but occurred with multiple foods.

Skin prick testing and serum-specific IgE testing to milk, egg, peanut, soy, and wheat were negative. Given ongoing parental concern and the unclear nature of the initial history, a supervised baked milk oral food challenge was performed. During the challenge, the child developed isolated unilateral erythema and sweating of the right cheek within 10 min of ingestion, without urticaria or systemic allergic features. The reaction resolved spontaneously without intervention. A video provided by the family, recorded at home during feeding with formula, demonstrated a similar unilateral cutaneous reaction.

Based on the characteristic distribution, reproducibility, absence of systemic involvement, and negative allergy testing, the diagnosis of Frey’s syndrome was made. Parents were reassured, dietary restrictions were lifted, and feeding-related anxiety improved.

## Discussion

Frey’s syndrome in infancy is rare but increasingly recognized. Dizon et al. [1] first described a series of eight infants presenting with localized flushing, often in the setting of perinatal trauma. Caulley et al. [2] documented an infant evaluated in family practice, highlghting the need for awareness beyond subspecialty clinics. Tillman et al. [3] described a 9-month-old with symptoms around the introduction of solids, while Hassan et al. [4] reported an infant repeatedly misdiagnosed with food allergy. Els and Delanty [5] highlighted another case termed “infantile Frey syndrome.” Finally, Betti et al. [6] systematically reviewed 121 non-surgical cases, demonstrating that pediatric presentations are not isolated curiosities but part of a broader spectrum.

Frey’s syndrome is thought to result from injury to parasympathetic fibers innervating the parotid gland, most commonly involving the auriculotemporal nerve [1]. During aberrant reinnervation, these parasympathetic fibers cross-innervate sympathetic pathways supplying cutaneous blood vessels and sweat glands in the periauricular and temporal region [1]. As a result, gustatory stimulation triggers localized flushing and sweating rather than salivation, producing a reproducible unilateral reaction that can be mistaken for food allergy.

The main diagnostic pitfall for allergists is assuming any reproducible food-triggered reaction must represent allergy. In infants, parental descriptions of facial flushing are often labeled as “hives,” contributing to misclassification as IgE-mediated allergy. As summarized in Table 1, IgE-mediated food allergy typically involves multi-organ symptoms such as urticaria, angioedema, wheeze, or vomiting, often with positive sensitization tests. By contrast, Frey’s syndrome is strictly unilateral, benign, and reproducible with diverse foods irrespective of allergenicity (Table 2).

**Table 1:** Distinguishing features of frey’s syndrome versus IgE-Mediated food allergy in infants

Feature	Frey’s syndrome	IgE-mediated food allergy
Onset with feeding	Yes, reproducible but non-allergen-specific	Yes, reproducible and allergen-specific
Distribution	Localized, unilateral, auriculotemporal nerve territory	Generalized or multi-system
Symptoms	Flushing, sweating	Urticaria, angioedema, respiratory, GI, cardiovascular signs
Systemic involvement	Absent	Common, multi-organ
IgE testing	Negative	Often positive
Course	Benign, non-progressive	Risk of anaphylaxis
Management	Reassurance, food reintroduction	Strict avoidance, emergency preparedness

**Fig. 1:**
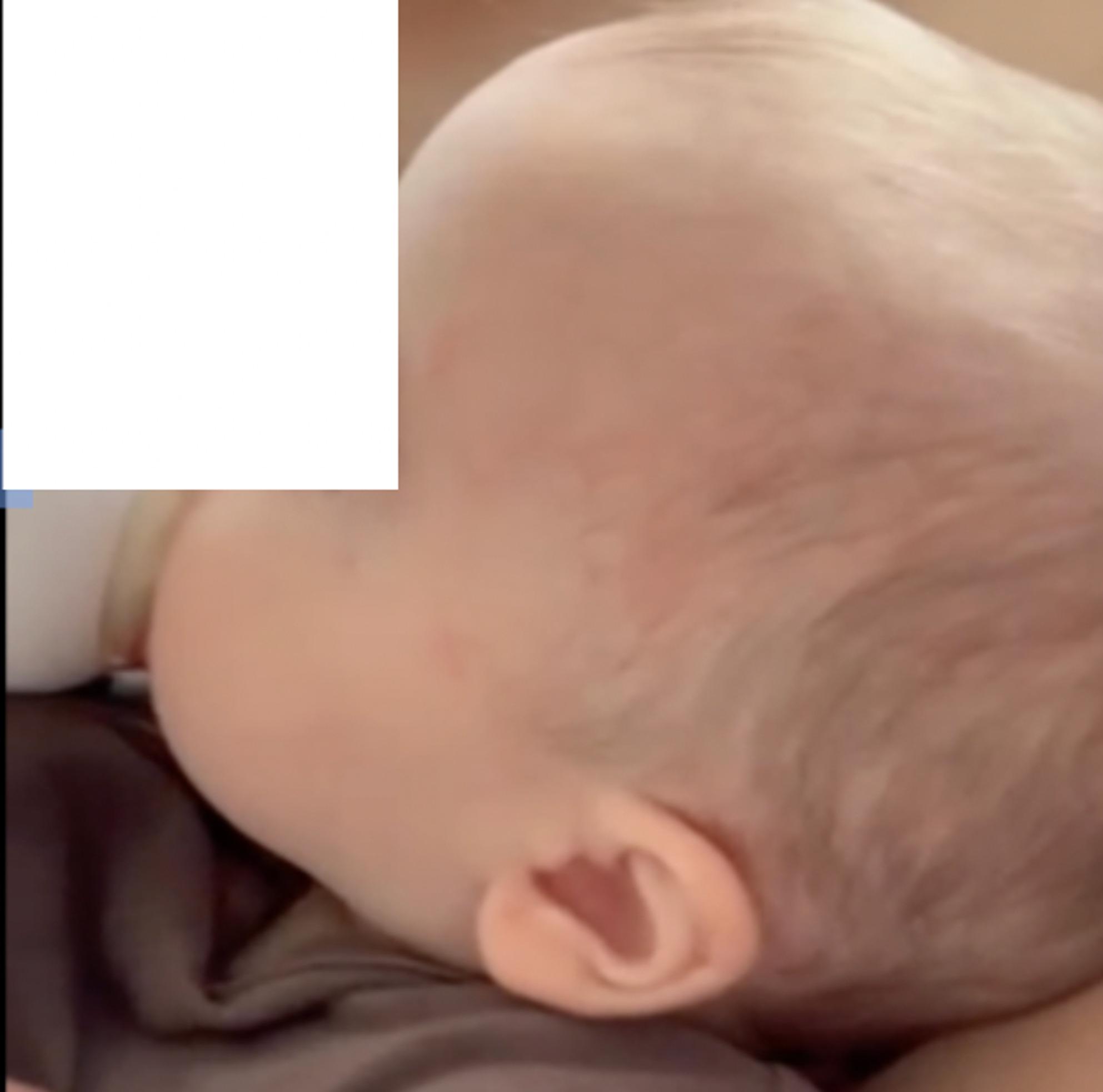
Home-recorded video demonstrating unilateral erythema and sweating on the right cheek during feeding with formula in an 8-month-old infant, consistent with Frey’s syndrome

Misdiagnosis carries meaningful consequences. Infants subjected to elimination diets risk impaired nutrition and growth, and avoidance of common foods may paradoxically promote development of true food allergy by interrupting oral tolerance acquisition. Families experience unnecessary anxiety and healthcare visits. Parental anxiety surrounding feeding reactions may further amplify concern for food allergy and contribute to escalation of testing (Fig. 1).

**Table 2:** Reported Pediatric/Infant frey’s (Auriculotemporal) syndrome cases and reviews

Source	Age at presentation	Trigger	Laterality	Key features	Outcome
Dizon et al. [1]	Infants (*n* = 8)	Feeding (various)	Unilateral	Flushing, occasional sweating; often forceps delivery	Benign; reassurance
Caulley et al. [2]	6 months	Chewing solids	Unilateral	Episodic flushing during meals; no systemic allergy signs	Reassurance
Tillman et al. [3]	9 months	Solids	Unilateral	Flushing ± sweating; mistaken for food allergy	Reassurance
Hassan et al. [4]	Infant (< 1 year)	Feeding	Unilateral	Recurrent erythema; no trauma; misdiagnosed as food allergy	Corrected diagnosis; foods reintroduced
Els and Delanty [5]	Infant	Feeding	Unilateral	“Infantile Frey syndrome”	Reassurance
Betti et al. [6]	Multiple pediatric cases	Feeding	Mostly unilateral	Systematic review of 121 non-surgical cases	Underrecognized; benign course

Management of infantile Frey’s syndrome consists primarily of reassurance and avoidance of unnecessary dietary restriction. Once recognized, referral to other specialties such as otolaryngology or neurology is typically not required. The condition is benign and often improves or resolves over time. Correct recognition allows reassurance, safe food reintroduction, and avoidance of iatrogenic harm.

Our case is notable for the combination of supervised clinical observation and home-recorded video documentation, which together helped distinguish Frey’s syndrome from IgE-mediated food allergy and facilitated diagnostic clarity for both clinicians and the family.

## Conclusion

Frey’s syndrome, though benign, is an important mimic of food allergy in infancy. This case illustrates how family anxiety, coupled with imprecise terminology in referral histories, can obscure recognition of benign feeding-related phenomena in infancy. For allergists, careful attention to laterality, distribution, and systemic features is essential. Awareness of this condition prevents misdiagnosis, protects nutritional status, and reassures families.

## Data Availability

All data generated or analyzed during this study are included in this published article.
